# Allelic variation of soybean flower color gene *W4* encoding dihydroflavonol 4-reductase 2

**DOI:** 10.1186/1471-2229-14-58

**Published:** 2014-03-06

**Authors:** Fan Yan, Shaokang Di, Felipe Rojas Rodas, Tito Rodriguez Torrico, Yoshinori Murai, Tsukasa Iwashina, Toyoaki Anai, Ryoji Takahashi

**Affiliations:** 1 Graduate School of Life and Environmental Sciences, University of Tsukuba, Tsukuba, Ibaraki 305-8518, Japan; 2 Department of Botany, National Museum of Nature and Science, Tsukuba, Ibaraki 305-0005, Japan; 3 Laboratory of Plant Genetics and Breeding, Faculty of Agriculture, Saga University, Honjo-machi, Saga 840-8502, Japan; 4 National Institute of Crop Science, Tsukuba, Ibaraki 305-8518, Japan

**Keywords:** Dihydroflavonol 4-reductase, Flavonoid, Flower color, *Glycine max*, *Glycine soja*, Soybean

## Abstract

**Background:**

Flower color of soybean is primarily controlled by six genes, viz., *W1*, *W2*, *W3*, *W4*, *Wm* and *Wp*. This study was conducted to investigate the genetic and chemical basis of newly-identified flower color variants including two soybean mutant lines, 222-A-3 (near white flower) and E30-D-1 (light purple flower), a near-isogenic line (Clark-*w4*), flower color variants (T321 and T369) descended from the *w4*-mutable line and kw4 (near white flower, *Glycine soja*).

**Results:**

Complementation tests revealed that the flower color of 222-A-3 and kw4 was controlled by the recessive allele (*w4*) of the *W4* locus encoding dihydroflavonol 4-reductase 2 (DFR2). In 222-A-3, a single base was deleted in the first exon resulting in a truncated polypeptide consisting of 24 amino acids. In Clark-*w4*, base substitution of the first nucleotide of the fourth intron abolished the 5′ splice site, resulting in the retention of the intron. The *DFR2* gene of kw4 was not expressed. The above results suggest that complete loss-of-function of *DFR2* gene leads to near white flowers. Light purple flower of E30-D-1 was controlled by a new allele at the *W4* locus, *w4-lp*. The gene symbol was approved by the Soybean Genetics Committee. In E30-D-1, a single-base substitution changed an amino acid at position 39 from arginine to histidine. Pale flowers of T369 had higher expression levels of the *DFR2* gene. These flower petals contained unique dihydroflavonols that have not yet been reported to occur in soybean and *G. soja*.

**Conclusions:**

Complete loss-of-function of *DFR2* gene leads to near white flowers. A new allele of the *W4* locus, *w4-lp* regulates light purple flowers. Single amino acid substitution was associated with light purple flowers. Flower petals of T369 had higher levels of *DFR2* gene expression and contained unique dihydroflavonols that are absent in soybean and *G. soja*. Thus, mutants of the *DFR2* gene have unique flavonoid compositions and display a wide variety of flower color patterns in soybean, from near white, light purple, dilute purple to pale.

## Background

**Figure 1:**
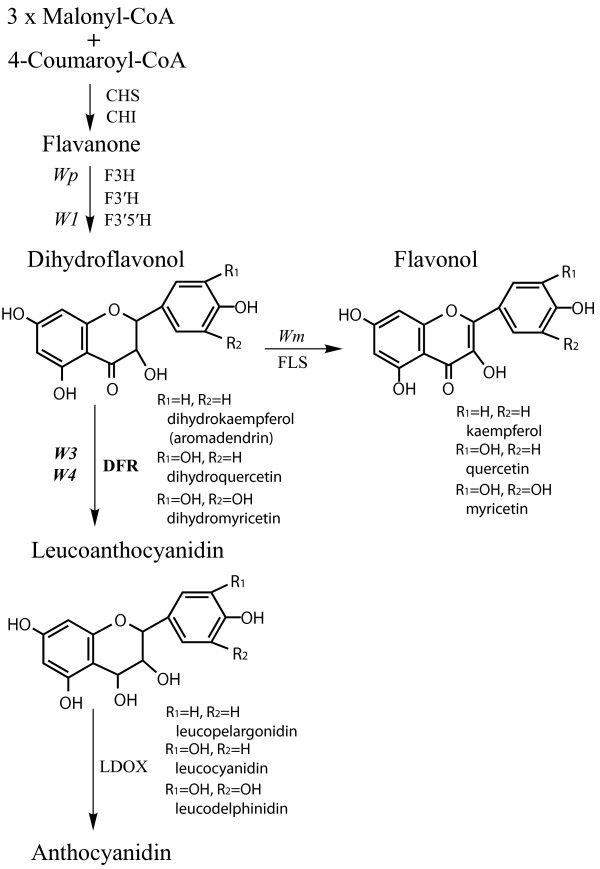
**Schematic diagram of the anthocyanin and flavonol biosynthetic pathways.** Enzyme names are abbreviated as follow: chalcone synthase (CHS), chalcone isomerase (CHI), flavanone 3-hydroxylase (F3H), dihydroflavonol 4-reductase (DFR), flavonoid 3′-hydroxylase (F3′H), flavonoid 3′5′-hydroxylase (F3′5′H), flavonol synthase (FLS), leucoanthocyanidin dioxygenase (LDOX). Soybean flower color genes encoding the enzymes are in italic font.

Flower color of soybean (*Glycine max* (L.) Merr.) is primarily controlled by six genes (*W1*, *W2*, *W3*, *W4*, *Wm* and *Wp*) [1,2]. Under *W1* genotype, soybean genotype with *W3W4* has dark purple, *W3w4* has dilute purple or purple throat, *w3W4* has purple, and *w3w4* has near white flowers [3]. Flower color of genotypes with allelic combination *W1w3w4* was indistinguishable from those with white flowers under many environments, suggesting that environments affect flower color under the allelic combination [3]. *W3* and *W4* encode dihydroflavonol 4-reductase (DFR) [4,5]. *W1*, *W2*, *Wm* and *Wp* encode flavonoid 3′5′-hydroxylase, MYB transcription factor, flavonol synthase and flavanone 3-hydroxylase, respectively [6-10]. The roles of these genes in the biosynthesis of anthocyanin and flavonol are presented in Figure 1.

The flavonoids in flower petals of soybean were analyzed [11-13]. The primary components of anthocyanin were malvidin 3,5-di-*O*-glucoside, petunidin 3,5-di-*O*-glucoside, delphinidin 3,5-di-*O*-glucoside and delphinidin 3-*O*-glucoside. In addition, eight flavonol glycosides, kaempferol 3-*O*-gentiobioside, kaempferol 3-*O*-rutinoside, kaempferol 3-*O*-glucoside, kaempferol 3-*O*-glycoside, kaempferol 3-*O*-rhamnosyl-(1→2)-[glucosyl-(1→6)-galactoside], kaempferol 7-*O*-glucoside, kaempferol 7-*O*-diglucoside and quercetin 3-*O*-gentiobioside, and one dihydroflavonol, aromadendrin 3-*O*-glucoside were identified. No anthocyanins were detected in Clark-*w1*, a near-isogenic line (NIL) of US cultivar Clark at the *W1* locus. Anthocyanins were not detected in a Clark-*w4* in 2003 and 2004, but trace amounts were detected in 2007 [11,12], indicating slight responsiveness to environmental conditions in agreement with the previous report [3].

**Figure 2:**
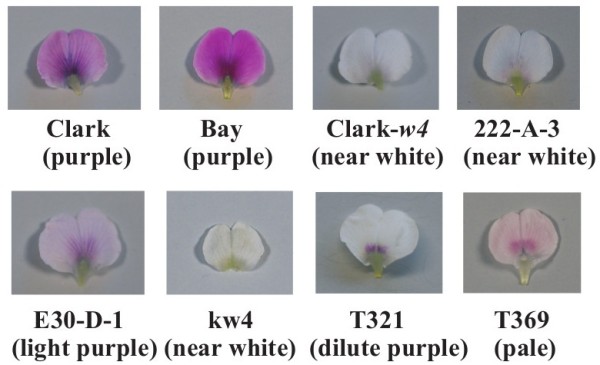
**Banner petals of flower color variants of soybean and *Glycine soja*.**

A mutable allele of the *W4* locus was discovered in a cross between two experimental lines with white and purple flowers, respectively [14]. The mutant line was designated as T322, and the mutable allele was designated as *w4-m*. Mutant lines T321 with *w4-dp* allele (dilute purple flower) and T369 with *w4-p* allele (pale flower) were isolated from descendants of T322 [15,16] (Figure 2). A 20.5-kb transposable element (*Tgm9*) was isolated from the second intron of the *DFR2* gene [5]. In T321 and T369, *Tgm9* was excised from the second intron, leaving behind 4- and 0-bp footprints, respectively [5]. A 5′ end fragment of *Tgm9* (944 bp) was integrated at a position 1043 bp upstream of the transcription start site in T321. A fragment of *Tgm9* was inserted at a position 1034 bp upstream of the transcription start site in T369. Soybean has two other *DFR* genes, *DFR1* and *DFR3*[17]. DNA marker analysis suggested that *W3* locus might correspond to the *DFR1*[17].

Flower color of the wild relative of soybean, *Glycine soja* Sieb. & Zucc. is almost exclusively purple; by contrast, 33% (5,544 out of 16,855) of the soybean accessions in the USDA Soybean Germplasm Collections have white flowers (Dr. R.L. Nelson, personal communication 2006). One white-flowered plant was found in 1998 among the progeny of a purple-flowered *G. soja* accession that was introduced from South Korea [18]. Genetic analysis indicated that the white flower was caused by a recessive allele at the *W1* locus similar to the white-flowered soybeans [18]. The mutation may have occurred during propagation at USDA. In 2002, a variant with light purple flowers, B09121 was discovered in southern Japan [13]. Genetic analysis suggested the light purple color was controlled by a new allele at the *W1* locus, *w1-lp*. Flower petals of B09121 contained lower amounts of the four major anthocyanins common in purple flowers and contained small amounts of the 5′-unsubstituted versions of the abovementioned anthocyanins, peonidin 3,5-di-*O*-glucoside, cyanidin 3,5-di-*O*-glucoside and cyanidin 3-*O*-glucoside [13]. B09121 may be the first example of a flower color variant of *G. soja* found in nature.

Lines varying in flower color were obtained from mutagenized populations of US cultivar Bay. Line 222-A-3 with near white flowers was isolated from an X-ray treated population, whereas line E30-D-1 with light purple flowers was developed from an EMS-treated population [19] (Figure 2). Dr. Donghe Xu (JIRCAS, Japan) found kw4, a *G. soja* accession with near white flowers, among accessions introduced from South Korea (personal communication, 2007) (Figure 2). It is unknown if the accession has near white flowers in the natural habitat. This study was conducted to investigate the genetic and chemical basis of flower color variants in *G. max* (Clark-*w4*, T321, T369, 222-A-3 and E30-D-1) and in *G. soja* (kw4).

## Methods

### Genetic analysis

The plant materials used in this study are listed in Table 1. US cultivars, Clark and Bay, have purple flowers (*W1W2w3W4WmWp*). Clark has tawny (*T*) and Bay has gray pubescence (*t*). 222-A-3 and E30-D-1 were crossed with Clark-*w4* (L68-1774, near white flower and tawny pubescence, *W1W2w3w4WmWpT*). E30-D-1 was also crossed with Clark. Flowers of 222-A-3 and E30-D-1 were emasculated one day before opening and fertilized with pollen from Clark or Clark-*w4*. A NIL of a Canadian cultivar Harosoy with *w4* allele, Harosoy-*w4* (L72-1138, near white flower and gray pubescence, *W1W2w3w4WmWpt*) was crossed with kw4 (tawny pubescence). Hybridity of the F_1_ plants was ascertained by tawny pubescence color. Seeds of NILs, T321 and T369 were provided by the USDA Soybean Germplasm Collection. The NILs were developed by backcrossing the near white flower trait six times from the cultivar Laredo into Clark or Harosoy (Table 1) [20].

**Table 1:** **Plant materials of soybean and *Glycine soja *** **used in this study**

**Line**	**Flower color**	**Genotype**	**Origin**	**Cross combination (Year of crossing)**
Clark	Purple	*W1W2w3W4WmWpT*	-	-
Bay	Purple	*W1W2w3W4WmWpt*	-	-
L68-1774 (Clark-*w4*)	Near white	*W1W2w3w4WmWpT*	L6^a^(6) × (Laredo × Harosoy)	-
L72-1138 (Harosoy-*w4*)	Near white	*W1W2w3w4WmWpt*	L2^b^(6) × Laredo	-
222-A-3	Near white	-	X-ray induced mutant of Bay	222-A-3 × Clark-*w4* (2007)
E30-D-1	Light purple	-	EMS-induced mutant of Bay	E30-D-1 × Clark (2012)
				E30-D-1 × Clark-*w4* (2008)
kw4	Near white	-	*G. soja* accession of South Korea	Harosoy-*w4* × kw4 (2008)
T321	Dilute purple	*W1W2w3w4-dpWmWpt*	Germinal revertant derived from T322	-
T369	Pale	*W1W2w3w4-pWmWpt*	Germinal revertant derived from T322	-

^a^A *Phytophtora* and pustle-resistant Clark isoline with genes *Rps1* and *rxp.*

^b^A *Phytophtora* and pustle-resistant Harosoy isoline with genes *Rps1* and *rxp.*

A total of seven F_1_ and 130 F_2_ seeds derived from Harosoy-*w4* × kw4 were field-planted on June 12 in 2009 at the National Institute of Crop Science, Tsukuba, Japan (36°06′N, 140°05′E). Similar numbers of F_1_ and F_2_ seeds derived from two crosses (222-A-3 × Clark-*w4* and E30-D-1 × Clark-*w4*) were planted on June 10 in 2010. A bulk of 30 seeds each of fifty F_3_ families derived from E30-D-1 × Clark-*w4* were planted on June 7 in 2012 and June 10 in 2013. A total of six F_1_ and 130 F_2_ seeds derived from E30-D-1 × Clark were planted on June 10 in 2013. N, P and K were applied at 3.0, 4.4 and 8.3 g m^-2^, respectively. Plants were individually grown with spacing of 70 cm between rows and 10 cm between plants. Flower color was recorded in individual F_1_, F_2_ and F_3_ plants.

### Analysis of flavonoids

Banner petals were collected at the day of opening from field-grown plants in 2008. Three 200 mg samples of banner petals were collected in 2 ml of MeOH containing 0.1% (v/v) HCl for anthocyanin analysis. Three 200 mg samples in 2 ml of absolute MeOH were also collected for the determination of flavonol and dihydroflavonol. High performance liquid chromatography (HPLC) of anthocyanins, flavonols and dihydroflavonol was performed following previously described protocols [11]. The 2 ml extracts were filtered through disposable filtration units (Maishoridisc H-13-5, Tosoh) and 10 μl from each sample was subjected to HPLC analysis. The amount of flavonoids was estimated from the pertinent peak area in the HPLC chromatogram (detection wavelength of anthocyanins = 530 nm; flavonols = 351 nm; dihydroflavonols = 290 nm). The peak area was subjected to analysis of variance using the Statistica software (StatSoft).

### Molecular cloning

Total RNA was extracted from banner petals (200 mg) using the TRIZOL Reagent (Invitrogen) according to the manufacturer’s instructions. cDNA was synthesized by reverse transcription of 5 μg of total RNA using the Superscript III First-Strand Synthesis System (Invitrogen) and an oligo(dT) primer according to the manufacturer’s instructions. The full-length cDNA was cloned by end-to-end PCR from the plant materials using a pair of PCR primers shown in Table 2. The PCR mixture contained 0.5 μg of cDNA, 10 pmol of each primer, 10 pmol of nucleotides and 1 unit of ExTaq in 1 × ExTaq Buffer supplied by the manufacturer in a total volume of 50 μl. A 5 min denaturation at 94°C was followed by 30 cycles of 30 sec denaturation at 94°C, 1 min annealing at 59°C and 1 min extension at 72°C. A final 7 min extension at 72°C completed the program. The PCR was performed in an Applied Biosystems 9700 thermal cycler. The PCR products were cloned into pCR 2.1 vector (Invitrogen) and sequenced. To evaluate the approximate size of PCR amplicons, PCR products were separated on a 2% agarose gel and visualized by EtBr staining.

**Table 2:** PCR primers used in this study

**Purpose**	**Target**	**Forward primer (5′-3′)**	**Reverse primer (5′-3′)**
cDNA cloning	*DFR2*	AACCAAAACAACGAGAGAGA	CTTATCCCTGATATGAAAGC
cDNA sequencing		TGCTAGACATCATGAAAGCA	TGTGAACAGCATATGTACCT
	*DFR2*	CACTGCTCTTTCACTAATCA	GATTAGTGAAAGAGCAGTGA
		TACCCTGAGTATAATGTCCT	TTCACGCATGCTTTCATGAT
Cloning of genomic fragment	Upstream fragment of *DFR2*	ACGGTTTCTTCCATTCCATT	ACTTGATTTCAGCCATGGTA
	Downstream fragment of *DFR2*	GTTCATCAATGCACATAGAC	CTTATCCCTGATATGAAAGC
Sequencing of genomic fragment	Upstream fragment of *DFR2*	TACAAGTTGTCATCACGATC	GAAGCTTTGATGAAGCCATT
		TTTGGTGTACACTCGTATGT	CACAATTATATCATTGGGCA
		ATGTAACATGATGGTTCGTG	AACCACCATTGCTTAATACC
	Downstream fragment of *DFR2*	CTTTTTCTCTGCAGGTTTCA	TAGTGGATGAATATGATTCT
		AAGTACCATTCCAACATTAA	GATAGATGACAGTTGTTGTC
		TGTTGTGCTCTTTGGCATAT	ACCCTGAGTATAATGTCCTT
CAPS analysis	*DFR2*	ACGGTTTCTTCCATTCCATT	CAAATGCTTCACCTTCTTCA
Cloning of 5′ upstream region	Upstream fragment of *DFR2*	AGAGATATATAAGAAGTTAGGA	TATCACGAAATAGTTTTTGTAAT
	Downstream fragment of *DFR2*	CCTTTACCATCTACAAGATAA	ATGATGTAATATTGGGAACCT
Sequencing of 5′ upstream region	*DFR2*	GAAAAGAGAAATAGGTATTATA	GTTTAACTAATCAAACTAAATT
Real-time PCR	*DFR2*	CCAAGGACCCTGAGAATGAA	CAGAAGTCAACATCGCTCCA
	*Actin*	GTCCTTTCAGGAGGTACAACC	CCACATCTGCTGGAAGGTGC

Genomic DNA was isolated from trifoliolate leaves by CTAB [21]. Genome sequences containing the entire coding region (about 3.3 kb) and the 5′ upstream region (about 1.2 kb) of Clark and kw4 were determined by cloning two fragments overlapping each other using the PCR primers listed in Table 2. The 5′ upstream region was also cloned from Bay and E30-D-1. The PCR mixture contained 10 ng of genomic DNA, 10 pmol of each primer, 10 pmol of nucleotides and 1 unit of ExTaq in 1 × ExTaq Buffer in a total volume of 50 μl. The PCR products were cloned into the pCR 2.1 vector.

### Sequencing analysis

Nucleotide sequences of both strands were determined with the BigDye terminator cycle method using an ABI3100 Genetic Analyzer (Applied Biosystems). Primers are exhibited in Table 2. Nucleotide sequences and the putative amino acid translations were analyzed with GENETYX ver. 8.1.2 (GENETYX). Sequences were aligned using ClustalW (http://clustalw.ddbj.nig.ac.jp/index.php?lang=ja) at default settings.

### CAPS analysis

Genomic DNA of Clark-*w4*, E30-D-1 and 40 F_2_ plants that were used for F_3_ progeny tests were isolated from trifoliolate leaves by CTAB. A pair of PCR primers (Table 2) was designed to detect a single-base substitution found in E30-D-1. The base substitution within the restriction site would result in the presence/absence of the restriction site of *Bsr*GI in the amplified product. The PCR mixture contained 30 ng of genomic DNA, 5 pmol of each primer, 10 pmol of nucleotides and 1 unit of ExTaq in 1 × ExTaq Buffer supplied by the manufacturer (Takara Bio) in a total volume of 25 μl. After an initial 30 sec denaturation at 94°C, there were 30 cycles of 30 sec denaturation at 94°C, 1 min annealing at 56°C and 1 min extension at 72°C. A final 7 min extension at 72°C completed the program. The amplified products were digested with *Bsr*GI, and the digests were separated on an 8% nondenaturing polyacrylamide gel in 1 × TBE buffer (90 mM Tris-borate, 2 mM EDTA, pH 8.0). After electrophoresis, the gel was stained with ethidium bromide and the DNA fragments were visualized under UV light.

### Quantitative real-time PCR

For quantitative real-time PCR, total RNA (5 μg) from each of three replicate banner petal samples was reverse-transcribed using the Superscript III First-Strand Synthesis System and an oligo d(T) primer. Primer sequences are exhibited in Table 2. The PCR mixture contained 0.4 μl of cDNA synthesis reaction mixture, 6 pmol of each primer, 1 × ROX reference dye, 1 × SYBR Premix Dimer Eraser (Takara Bio) and water to a final volume of 20 μl. Analysis was done using the StepOnePlus Real-Time PCR System (Applied Biosystems). The initial 30 sec denaturation at 95°C was followed by 40 cycles of 3 sec denaturation at 95°C, 30 sec annealing at 58°C and 30 sec extension at 72°C. The expression level of the soybean *actin* gene (GenBank accession number: J01298) [22] was used to normalize target gene expression.

### Accession numbers

Sequence data of the *DFR2* gene were deposited in the DDBJ Data Libraries under accession nos. AB872212 (cDNA of Bay), AB872213 (cDNA of Clark-*w4*), AB872214 (cDNA of 222-A-3), AB872215 (cDNA of E30-D-1), AB872216 (genomic DNA of Clark) and AB872217 (genomic DNA of kw4).

## Results

### Genetic analysis

F_1_ plants derived from a cross between Harosoy-*w4* and kw4 had near white flowers (Table 3). All of the 116 plants of the F_2_ population had near white flowers, suggesting that flower color of kw4 was controlled by the *w4* allele. F_1_ plants derived from a cross between 222-A-3 and Clark-*w4* had near white flowers. All of the 109 plants of the F_2_ population had near white flowers, suggesting that flower color of 222-A-3 was also controlled by the *w4* allele.

**Table 3:** **Segregation of flower color in F**_**1 **_**plants and F**_**2 **_**populations derived from crosses between soybean cultivar Clark or near-isogenic lines (Harosoy-*w4 *** **and Clark- *w4 *) and flower color variants, *Glycine soja *** **accession, kw4, and soybean mutant lines, 222-A-3 and E30-D-1 in Tsukuba, Japan**

**Generation**	**Year**	**Number of plants**				**Expected ratio**	**χ**^**2 **^**value**	**Probability (*P *** **value)**
		**Total**	**Purple**	**Light purple**	**Near white**			
kw4	2009	10	-	-	10	-	-	-
Harosoy-*w4* (H-*w4*)	2009	10	-	-	10	-	-	-
H-*w4* × kw4 F_1_	2009	5	-	-	5	-	-	-
H-*w4* × kw4 F_2_	2009	116	-	-	116	-	-	-
222-A-3 (222)	2010	10	-	-	10	-	-	-
Clark-*w4* (C-*w4*)	2010	10	-	-	10	-	-	-
222 × C-*w4* F_1_	2010	5	-	-	5	-	-	-
222 × C-*w4* F_2_	2010	109	-	-	109	-	-	-
E30-D-1 (E)	2010	10	-	10	-	-	-	-
Clark (C)	2010	10	10	-	-	-	-	-
E × C F_1_	2010	4	4	-	-	-	-	-
E × C F_2_	2013	112	84	28	-	3:1	0.00	1.00
E × C-*w4* F_1_	2010	3	-	3	-	-	-	-
E × C-*w4* F_2_	2010	111	-	82	29	3:1	0.08	0.78

F_1_ plants derived from a cross between E30-D-1 and Clark had purple flowers. A total of 112 plants of the F_2_ population segregated into 84 plants with purple flowers and 28 plants with light purple flowers. The segregation fitted a 3:1 ratio (χ^2^ = 0.00, P = 1.00) suggesting that a single gene controls flower color and that the allele for purple flower was dominant to that for light purple flower. F_1_ plants derived from a cross between E30-D-1 and Clark-*w4* had light purple flowers. A total of 111 plants of the F_2_ population segregated into 82 plants with light purple flowers and 29 plants with near white flowers. The segregation fitted a 3:1 ratio (χ^2^ = 0.08, P = 0.78) suggesting that the *W4* locus controls the flower color and the allele for light purple flower was dominant to that for near white flower. All of the ten F_3_ families derived from F_2_ plants with near white flowers had near white flowers. A total of 40 families derived from F_2_ plants with light purple flowers segregated into 16 families fixed for light purple flowers and 24 families segregating for flower color (Table 4). The segregation fitted a 1:2 ratio (χ^2^ = 0.80, P = 0.37) confirming that an allele at the *W4* locus controls flower color. The new allele was designated as *w4-lp*. The gene symbol was approved by the Soybean Genetics Committee. The dominance relationship of the alleles is *W4* > *w4-lp* > *w4*.

**Table 4:** **Segregation of flower color in F**_**3 **_**families derived from a cross between E30-D-1 and a soybean near-isogenic line Clark-*w4 *** **in 2012 and 2013 in Tsukuba, Japan**

**Line**	**Number of families**				**Expected ratio**	**χ**^**2 **^**value**	**Probability (*P *** **value)**
	**Total**	**Fixed for light purple**	**Segregating**	**Fixed for near white**			
E30-D-1 × Clark-*w4* F_3_ (light purple)^*a*^	40	16	24	-	1:2	0.80	0.37
E30-D-1 × Clark-*w4* F_3_ (near white)^*b*^	10	-	-	10	-	-	-

^*a*^F_3_ families derived from F_2_ plants with light purple flowers.

^*b*^F_3_ families derived from F_2_ plants with near white flowers.

### HPLC analysis

Four anthocyanin components, A1: malvidin 3,5-di-*O*-glucoside, A2: petunidin 3,5-di-*O*-glucoside, A3: delphinidin 3,5-di-*O*-glucoside, A4: delphinidin 3-*O*-glucoside were detected in agreement with previous studies [11,12] (Table 5). Flowers of T369 contained 59.8% of total anthocyanins compared with Clark. Less anthocyanins were detected in T321 (44.7%) and E30-D-1 (39.3%). Near white flowers of 222-A-3 had the lowest level of anthocyanins (15.6%). Near white flowers of kw4 had only trace amount of the two components, A1 and A2. All cultivars and lines except for 222-A-3 and kw4 had all four components with the amounts decreasing in the following order: A1 > A2 > A3 > A4.

**Table 5:** **Anthocyanin content [mean ± SD (× 10**^**3**^**)] according to HPLC analysis of flower petals from soybean and *Glycine soja *** **in 2008 at Tsukuba, Japan**

**Line name**	**A1**^**a**^	**A2**	**A3**	**A4**	**Total**
Clark	933 ± 40	538 ± 21	399 ± 13	255 ± 20	2,125 ± 77
Bay	1,745 ± 326	610 ± 99	323 ± 62	267 ± 14	2,945 ± 331
222-A-3	142 ± 28	189 ± 30	0 ± 0	0 ± 0	331 ± 16
E30-D-1	345 ± 4	241 ± 20	151 ± 3	98 ± 65	835 ± 65
kw4	t^b^	t	0 ± 0	0 ± 0	-
T321	369 ± 107	255 ± 22	178 ± 36	148 ± 4	950 ± 155
T369	513 ± 32	348 ± 17	232 ± 44	178 ± 8	1,271 ± 89
LSD_0.05_	239	76	58	48	268

^a^A1: malvidin 3,5-di-*O*-glucoside, A2: petunidin 3,5-di-*O*-glucoside, A3: delphinidin 3,5-di-*O*-glucoside, A4: delphinidin 3-*O*-glucoside.

^b^Trace amount.

All cultivars and lines had eight flavonol glycoside components, F1 (kaempferol 3-*O*-gentiobioside), F2 (kaempferol 3-*O*-rutinoside), F3 (kaempferol 3-*O*-glucoside), F4 (kaempferol 3-*O*-glycoside), F5 (kaempferol 3-*O*-rhamnosyl-(1 → 2)-[glucosyl-(1 → 6)-galactoside]), F6 (quercetin 3-*O*-gentiobioside), F7 (kaempferol 7-*O*-glucoside), F8 (kaempferol 7-*O*-diglucoside) in accordance with previous studies [11-13] (Table 6). The total amounts of flavonol glycosides were not very different among cultivars and lines except for T369. F1 was most abundant and accounted for about 80% of flavonol glycosides in these cultivars and lines in accordance with previous studies [11,12]. The amount of F2 was extremely low in kw4 and comprised only 0.1% of the total amount of flavonol glycosides. Flowers of T369 had substantially lower amount of flavonol glycosides (16.0% of Clark).

**Table 6:** **Flavonol glycoside content [mean ± SD (× 10**^**3**^**)] according to HPLC analysis of flower petals from soybean and *Glycine soja *** **in 2008 at Tsukuba, Japan**

**Line name**	**F1**^**a**^	**F2**	**F3**	**F4**	**F5**	**F6**	**F7**	**F8**	**Total**
Clark	9,432 ± 103	772 ± 34	177 ± 6	441 ± 26	353 ± 38	138 ± 10	13 ± 0	128 ± 13	11,454 ± 177
Bay	8,508 ± 278	788 ± 52	162 ± 5	246 ± 10	429 ± 26	131 ± 8	53 ± 0	179 ± 15	10,496 ± 385
222-A-3	7,836 ± 426	698 ± 39	134 ± 9	275 ± 33	459 ± 42	124 ± 2	323 ± 55	365 ± 15	10,214 ± 477
E30-D-1	8,001 ± 491	732 ± 37	168 ± 78	335 ± 24	465 ± 20	117 ± 32	274 ± 43	318 ± 56	10,409 ± 630
kw4	10,947 ± 386	16 ± 2	432 ± 5	695 ± 26	802 ± 50	154 ± 7	354 ± 52	417 ± 38	13,816 ± 533
T321	9,417 ± 476	805 ± 50	159 ± 17	371 ± 20	523 ± 16	100 ± 2	174 ± 10	287 ± 12	11,838 ± 598
T369	703 ± 9	214 ± 26	102 ± 2	135 ± 1	243 ± 5	151 ± 2	130 ± 6	158 ± 16	1,837 ± 55
LSD_0.05_	647	69	56	41	58	24	60	52	827

^a^F1 (kaempferol 3-*O*-gentiobioside), F2 (kaempferol 3-*O*-rutinoside), F3 (kaempferol 3-*O*-glucoside), F4 (kaempferol 3-*O*-glycoside), F5 (kaempferol 3-*O*-rhamnosyl-(1→2)-[glucosyl-(1→6)-galactoside]), F6 (quercetin 3-*O*-gentiobioside), F7 (kaempferol 7-*O*-glucoside), F8 (kaempferol 7-*O*-diglucoside).

**Figure 3:**
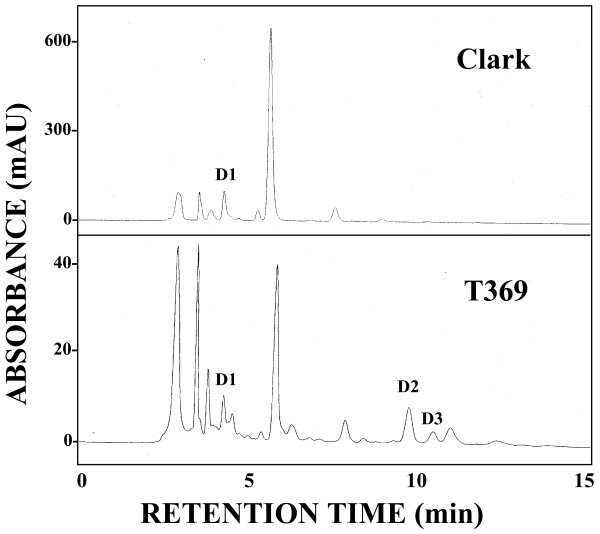
**HPLC chromatogram of dihydroflavonols extracted from flower petals of a soybean cultivar Clark and T369.** A total of 200 mg of banner petals was extracted with 2 ml of MeOH. Eluents: MeCN/H_2_O/H_3_BO_3_  (22:78:0.2). Flow-rate: 1.0 ml/min. Injection: 10 μl. Detection: 290 nm. D1, aromadendrin 3-*O*-glucoside; D2 and D3, unidentified dihydroflavonols.

Only one kind of dihydroflavonol (D1, aromadendrin 3-*O*-glucoside) was detected in all cultivars and lines except for T369 (Table 7). The amount varied from 57.4 (E30-D-1) to 163.8% (kw4) compared with Clark. In contrast, flower petals of T369 contained only 11.4% of D1 compared with Clark, in addition to two unique peaks corresponding to dihydroflavonols, D2 and D3 (Figure 3).

**Table 7:** **Dihydroflavonol content [mean ± SD (× 10**^**3**^**)] according to HPLC analysis of flower petals from soybean and *Glycine soja *** **in 2008 at Tsukuba, Japan**

**Line name**	**D1**^**a**^	**D2**^**b**^	**D3**^**b**^	**Total**
Clark	843 ± 53	0 ± 0	0 ± 0	843 ± 53
Bay	758 ± 83	0 ± 0	0 ± 0	758 ± 83
222-A-3	593 ± 40	0 ± 0	0 ± 0	593 ± 40
E30-D-1	484 ± 24	0 ± 0	0 ± 0	484 ± 24
kw4	1,381 ± 58	0 ± 0	0 ± 0	1,381 ± 58
T321	646 ± 22	0 ± 0	0 ± 0	646 ± 22
T369	96 ± 8	153 ± 39	54 ± 10	303 ± 54
LSD_0.05_	86	27	7	94

^a^D1: aromadendrin 3-*O*-glucoside.

^b^D2, D3: unidentified dihydroflavonols.

### Molecular cloning

**Figure 4:**
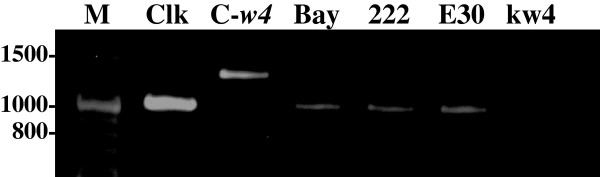
**Agarose gel electrophoresis of RT-PCR products corresponding to the entire coding region of *DFR2 *gene in soybean and *Glycine soja*.** M, 100 bp ladder marker; Clk, Clark; C-*w4*; Clark-*w4*; 222, 222-A-3; E30, E30-D-1. The migration of size markers (bp) is shown to the left of the gel.

**Figure 5:**
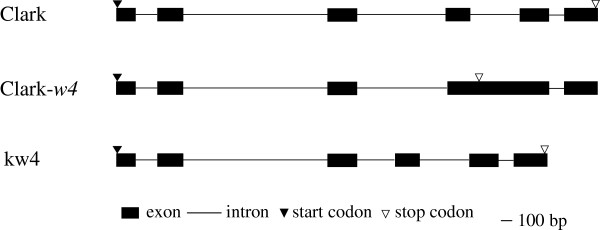
**Intron/exon structure of *DFR2 *** **gene from Clark (*G. max*), a Clark near-isogenic line with *w4 *** **allele, Clark-*w4 *** **and kw4, a *Glycine soja *** **accession.**

DNA fragments of about 1.1 kb were amplified by RT-PCR in Clark, Bay, 222-A-3 and E30-D-1 (Figure 4). Fragments of about 1.4 kb were amplified from Clark-*w4*. No amplification product was observed in kw4. The coding region of *DFR2* gene of Clark and Bay were 1065 bp long and they encoded 354 amino acids. Amino acids were identical except for two substitutions around the C-terminus at positions 338 (valine or glutamic acid) and 353 (arginine or glutamine). Comparison of nucleotide sequences between cDNA and genomic DNA of Clark revealed that the *DFR2* gene has six exons and five introns similar to a previous report (Figure 5) [5].

**Figure 6:**
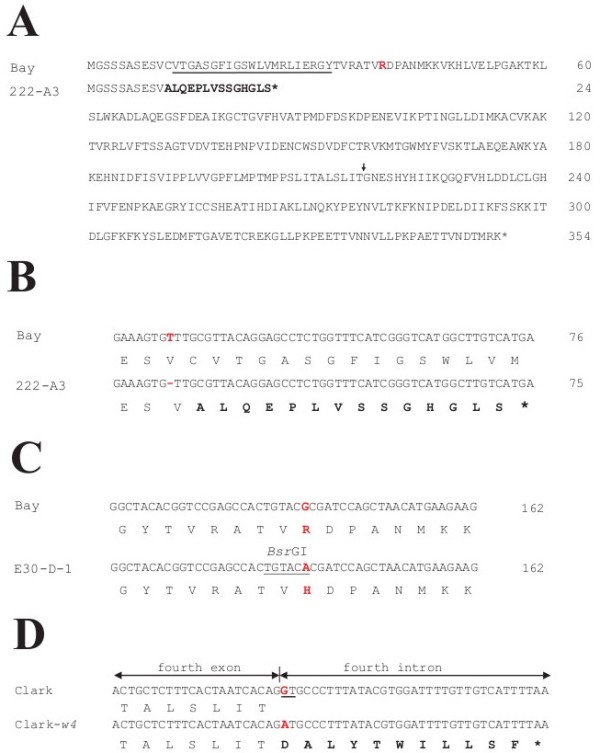
**Nucleotide and amino acid polymorphisms of the *DFR2 *gene in flower color variants of soybean. (A)** Amino acid sequence of Bay and 222-A-3. Amino acids polymorphic in 222-A-3 are shown in bold. NADPH binding domain is underlined. The polymorphic amino acid in E30-D-1 is shown in red font. End of the fourth exon is indicted by an arrow. **(B)** Alignment of partial cDNA and amino acid sequences from Bay and 222-A-3. Polymorphic nucleotides are shown in red font. The polymorphic amino acids in 222-A-3 are shown in bold. **(C)** Alignment of partial cDNA and amino acid sequences from Bay and E30-D-1. Polymorphic nucleotides and amino acids are shown in red font. Restriction site used for CAPS analysis is underlined. **(D)** Alignment of partial nucleotide and amino acid sequences around the end of the fourth exon of Clark and the corresponding region of Clark-*w4*. Polymorphic nucleotides are shown in red font. Nucleotides common in the 5′ splice site are underlined. Amino acids polymorphic in Clark-*w4* are shown in bold.

Bay had a T at nucleotide position 29 which was absent in 222-A-3. This deletion probably generated a truncated polypeptide consisting of only 24 amino acids (Figure 6A and 6B). The polypeptide lacked the NADPH binding domain [23]. In E30-D-1, a single base was substituted from G to A at nucleotide position 116 compared with Bay (Figure 6C). The base-substitution altered amino acid at position 39 from arginine to histidine. The 5′ upstream region of E30-D-1 was identical with that of Bay and Clark. In Clark-*w4*, cDNA had a 344-bp insertion compared with Clark and Bay. The insertion corresponded to the fourth intron with five nucleotide substitutions compared with Clark, suggesting that the fourth intron was retained in Clark-*w4*. In Clark-*w4*, a single-base G at the start of the fourth intron was changed to A compared with the genome sequence of Clark (Figure 6D). The base substitution may have abolished the 5′ splice site (GT) resulting in the retention of the intron (Figure 5). The retention caused a mutation from amino acid position 217 and premature translation termination at amino acid position 227 (Figure 6D).

In kw4, transcripts of the *DFR2* gene in the flower petals were not detected by RT-PCR. The genomic fragment containing the entire coding region was amplified by PCR. Six exons and five introns were assumed similar to Clark (Figure 5). The amino acid sequence was identical with that of Clark. A 367-bp fragment was deleted in the third intron of kw4 (Figure 5). The 5′ upstream region of kw4 had six single-base substitutions, three single-base indels, two two-base indels and a three-base alteration including one indel (Additional file 1: Figure S1).

### CAPS analysis

**Figure 7:**
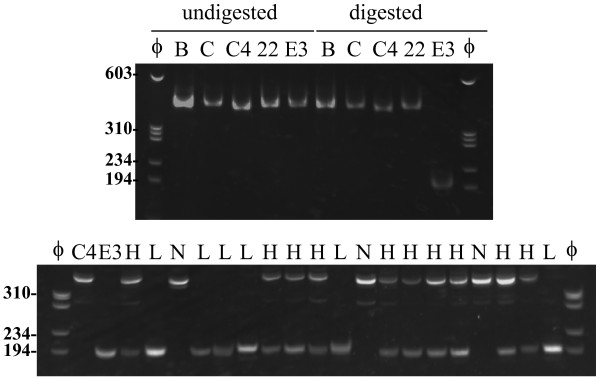
**Results of CAPS analysis of *DFR2 *gene in soybean.** (Upper panel) Results of CAPS analysis for flower color variants. PCR products amplified with CAPS primers were digested by *Bsr*GI and the digests were separated on an 8% polyacrylamide gel. ф, molecular marker фx174/*Hae*III; B, Bay; C, Clark; C4, Clark-*w4*; 22, 222-A3; E3, E30-D-1. (Lower panel) Results of CAPS analysis in an F_2_ population derived from a cross between E30-D-1 and Clark-*w4*. ф, фx174/*Hae*III; C4, Clark-*w4*; E3, E30-D-1; H, F_2_ plants segregating for flower color; L, F_2_ plants fixed for light purple flower; N, F_2_ plants fixed for near white flower. The migration of size markers is shown to the left of the gel.

PCR with CAPS primers generated amplified products of 377 bp in Bay, Clark, Clark-*w4*, 222-A-3 and E30-D-1 (Figure 7). Digestion with *Bsr*GI generated a band of 194 bp in E30-D-1, whereas the bands were not digested in the other materials (Figure 7). CAPS analysis of an F_2_ population derived from a cross between E30-D-1 and Clark-*w4* together with the F_3_ progeny tests revealed that plants fixed with light purple flowers had only a shorter band, plants fixed with near white flowers had only a longer band and plants segregating for flower color had both bands. Thus, the CAPS marker co-segregated with flower color.

### Quantitative real-time PCR

**Figure 8:**
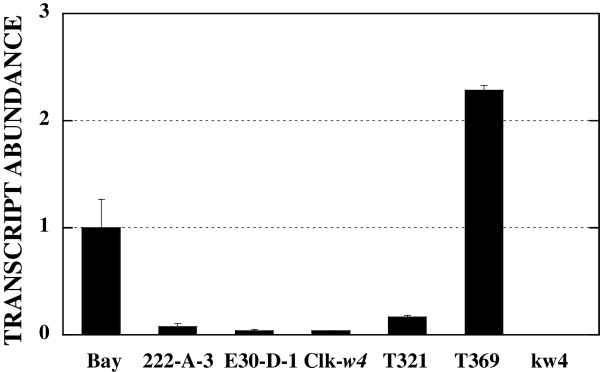
**Expression of *DFR2 *gene relative to the cultivar Bay in flower petals of soybean and *Glycine soja*.** Transcript levels were standardized to the transcript level of actin. The means and SDs from three biological replications are exhibited.

Results of real-time PCR are presented in Figure 8. Transcript level of T321 was low and 16.8% of Bay. Transcript levels of 222-A-3, E30-D-1 and Clark-*w4* were much lower at 7.7, 3.8 and 3.7%, respectively. Transcripts of the *DFR2* gene of kw4 were not detected by real-time PCR. In contrast to the above flower color variants, the transcript level of T369 was about 2.3 times of Bay (Figure 8).

## Discussion

Previous studies revealed that the *W4* gene was mutated in flower color variants, Clark-*w4*, T321 and T369 [3,5]. In this study, complementation tests revealed that the flower color of 222-A-3 and E30-D-1, and that of a *G. soja* accession, kw4, was also controlled by this gene. Amino acid polymorphism or null expression of the *DFR2* gene was associated with flower color variation.

In 222-A-3, a single-base deletion caused a frame-shift mutation from amino acid position 11; this is expected to produce a truncated polypeptide of only 24 amino acids that lacked the NADPH binding domain. Thus, the *DFR2* transcript of 222-A-3 may be nonfunctional. In Clark-*w4*, the first nucleotide of the fourth intron was substituted from G to A. The base substitution in Clark-*w4* may have abolished the 5′ splice site (spliceosome recognition site). Retention of the fourth intron caused a frame-shift mutation and changed subsequent amino acids. Translation was prematurely terminated at amino acid position 227. *DFR* genes have many amino acids conserved across plant species in the downstream of the mutation [24]. The results strongly suggest that the *DFR2* gene of Clark-*w4* is not functioning. In kw4, the *DFR2* gene was not expressed in flower petals. A 367-bp fragment was deleted from the third intron of this gene in kw4. However, it is not clear whether the deletion in the intron might be responsible for null gene expression. Therefore, we investigated the 5′ upstream region to check if any mutation occurred in the promoter region. There were many nucleotide polymorphisms in the 5′ upstream region; six single-base substitutions, three single-base indels, two two-base indels and a three-base alteration including an indel. The accumulation of a substantial number of mutations in the promoter region, which is probably the reason for non-transcription of this locus in kw4, is characteristic of genes that are being deactivated into pseudogenes. Promoter assays may be necessary to determine which polymorphism is critical for gene expression. Features of DNA sequences in Clark-*w4*, 222-A-3 and kw4 strongly suggest that complete loss-of-function of *DFR2* gene may lead to substantial reduction of anthocyanins and near white flowers.

Soybean has three variants of *DFR* genes, *DFR1*, *DFR2* and *DFR3*[17]. The function of *DFR2* may be partially supplemented by the activity of other *DFR* genes depending on environmental conditions. The transcript level of Clark-*w4* and 222-A-3 was substantially lower than that of Bay, probably because of nonsense-mediated mRNA decay, a surveillance mechanism to eliminate aberrant mRNA transcripts that contain premature stop codons [25].

The 5′ upstream regions of the *DFR2* gene in E30-D-1, Bay and Clark were identical. In the first exon of E30-D-1, however, a single-base substitution altered an amino acid at position 39 from arginine to histidine. The position of the residue was slightly downstream of the NADPH binding region. No catalytic domain has been assigned to the region, but the arginine residue is conserved across eight plant species [24]. Further, CAPS marker to detect the base substitution co-segregated with flower color. These results suggest that the amino acid substitution might have affected transcript abundance and/or DFR function resulting in reduced anthocyanin contents and paler flower color. Transgenic experiments may be necessary to ascertain the functional importance of this residue.

Flavonol glycoside content in flower petals of T321 was similar to that of Clark. In contrast, that of T369 was 16.0% of Clark. The *DFR2* gene was over-expressed in flower petals of T369 but it was barely expressed in T321. The reduction of flavonol glycosides in T369 may be explained by substrate competition between over-expressed DFR and flavonol synthase (Figure 1). Flower petals of T369 contained substantially lower amounts of D1 but it had unique dihydroflavonol components, D2 and D3, that are absent in the soybean and *G. soja* accessions analyzed so far. Over-expression of *DFR2* gene may be responsible for the unique dihydroflavonol composition. Chemical structure of D2 and D3 should be determined to investigate the novel features of DFR2 function displayed by T369.

In T321 and T369, *Tgm9* was excised from the second intron of the *DFR2* gene, leaving behind 4- and 0-bp footprints, respectively [5]. A 5′ end fragment of *Tgm9* (944 bp) was integrated at the 1043 bp upstream of the transcription start site in T321. A fragment of *Tgm9* was inserted at the 1034 bp upstream of the transcription start site in T369 [5]. In both cases, excision of *Tgm9* may not be the cause of flower color change, because *Tgm9* resides in the intron, and footprints, if any, are not likely to substantially affect gene expression. Instead, re-insertion into the promoter region is more likely responsible. The upstream promoter regions of structural anthocyanin biosynthesis genes contain cis regulatory elements that affect pigmentation patterns or intensity [5]. It is interesting to determine the role of 9-bp differences in the *Tgm9* integration site in the expression of the *DFR2* gene. Detailed promoter assays may be necessary to identify the cis element regulating the expression of this gene.

*DFR2* gene of soybean controls intensity and distribution of pigmentation in flower petals. Mutation of the gene results in unique flavonoid composition and a wide variety of flower color patterns, from near white, light purple, dilute purple to pale.

## Conclusions

The flower colors of 222-A-3, Clark-*w4*, E30-D-1, kw4, T321 and T369 were controlled by the *W4* gene encoding DFR2. In 222-A-3, a single-base deletion probably produced a truncated polypeptide consisting of 24 amino acids. In Clark-*w4*, base substitution of the first nucleotide of the fourth intron abolished the 5′ splice site, resulting in the retention of the intron. The *DFR2* gene of kw4 was not expressed. The above results suggest that complete loss-of-function of *DFR2* gene leads to near white flowers. Flower color of E30-D-1 was controlled by a new allele of the *W4* locus, *w4-lp*. In E30-D-1, a single-base substitution changed an amino acid at position 39 from arginine to histidine. In T369, expression of *DFR2* gene was 2.3 times that of purple flowers, and the flower petals contained unique dihydroflavonols which are absent in other *G. max* and *G. soja* accessions. Thus, mutations of *DFR2* gene results in unique flavonoid compositions and a wide variety of flower color patterns in soybean, ranging from near white, light purple, dilute purple to pale.

## Abbreviations

CHI: Chalcone isomerase; CHS: Chalcone synthase; DFR: Dihydroflavonol 4-reductase; F3H: Flavanone 3-hydroxylase; F3′H: Flavonoid 3′-hydroxylase; F3′5′H: Flavonoid 3′5′-hydroxylase; FLS: Flavonol synthase; G. soja: *Glycine soja*; HPLC: High performance liquid chromatography; LDOX: Leucoanthocyanidin dioxygenase; NIL: Near-isogenic line.

## Competing interests

The authors declare that they have no competing interests.

## Authors’ contributions

RT designed and supervised the research. YM and TI performed chemical analysis. TA discovered and provided novel mutant lines. FY and SD performed molecular biology experiments. RT, TA, FRR and TRT performed genetic analysis. All authors read and approved the final manuscript.

## Supplementary Material

Additional file 1: Figure S1 Alignment of the 5′ upstream region of *DFR2* gene in soybean cultivar Clark and a *Glycine soja* accession kw4. Polymorphic nucleotides are shown in red font. Coding sequence is underlined. Click here for file
