# Supplementary material for: Allelic variation of soybean flower color gene W4 encoding dihydroflavonol 4-reductase 2
Source: BMC Plant Biol. 2014 Mar 6;14:58. doi: 10.1186/1471-2229-14-58 (PMC4015899; doi:10.1186/1471-2229-14-58)
Supplement: Additional file 1: Figure S1 — Alignment of the 5′ upstream region of DFR2 gene in soybean cultivar Clark and a Glycine soja accession kw4. Polymorphic nucleotides are shown in red font. Coding sequence is underlined. [file 1471-2229-14-58-S1.pdf]

```
Clark   GAGATATATAAGAAGTTAGGAACAAAAATGAAGGATGAAATGTGTGAGCAGTCAGTGAGC -1157
kw4     GAGATATATAAGAAGTTAGGAACAAAAATGAAGGATGAAATGTGTGAGCAGTCAGTGAGC -1160

Clark   CACCAATCAGCAGTCACGTGCTCTTTAGTTCAGCTAACTTGTTGTACCTAACATAACCGG -1097
kw4     CACCAATCAGCAGTCACGTGCTCTTTAGTTCAGCTAACTTGTTGCACCTAACATAACCGG -1100

Clark   CTGGTGATCACGAGTTGAGTAAAAGACAGGTATCATAAAATAACACAATGAAATAATAAT -1037
kw4     CTGGTGATCACGGGTTGAGTAAAAGACAGGTATCATAAAATAACACAATGAAATAATAAT -1040

Clark   GAACCAATTTCCATAAAAGATGTGCTTCGATGTGGACCTGCATTGTCTTTGTCCTATGCC  -977
kw4     GAACCAATTTCCATAAAAGATGTGCTTCGATGTGGACCTGCATTGTCTTTGTCCTATGCC  -980

Clark   ATGTGAGAATAAAGCAGATTTTAATTTGGAATTTCCTAGTTTAAAATAGCACATGTTTTC  -917
kw4     ATGTGAGAATAAAGCAGATTTTAATTTGGAATTTCCTAGTTTAAAATAGCACATGTTTTC  -920

Clark   ATATTAATATTTTTAAAATGTTAATTAAAATAGATTTTTCAAATATTTCATTTATCAGTA  -857
kw4     ATATTAATATTTTTAAAATGTTAATTAAAATAGATTTTTCAAATATTTCATTTATCAGTA  -860

Clark   TGAATGTATTTTTCTTAAAAACCTTAATGAGTCATCTGTATGCATGACACCATATTAGTT  -797
kw4     TGAATGTATTTTTCTTAAAAACCTTAATGAGTCATCTGTATGCATGACACCATATTAGTT  -800

Clark   TTTTATAA-AAAAAAAAATCAATAAGCTAGTATCTTATCCTTTACCATCTACAAGATAAT  -738
kw4     TTTTTTTTTAAAAAAAAATCAATAAGCTAGTATTTTATCCTTTACCATCTACAAGATAAT  -740

Clark   TTTGTTTTATTTAACTAAA-TCTATAATAAATAAATATTTTTTTAACATATAAATTATGT  -679
kw4     TTTGTTTTATTTAACTAAAATCTATAATAAATAAATATTTTTTTAACATATAAATTATGT  -680

Clark   TATAATTAAAATTTCTAATAAATAAATATTTTTTATATATAAATTATATTGAAATTTATT  -619
kw4     TATAATTAAAATTTCTAATAAATAAATATTTTTTATATAAAAATTATATTGAAATTTATT  -620

Clark   ACAAAAACTATTTCGTGATAATAAAATTACTTTTAATTATTGATACTCTTTTAAAAAA-T  -560
kw4     ACAAAAACTATTTCGTGATAATAAAATTACTTTTAATTATTGATACTCTTTTAAAAAAAT  -560

Clark   ATAAAAAATTAAATTCAAAAATAAAAATGAAAATAATAGATTTAATAAGATAAGAGGTCA  -500
kw4     ATAAAAAATTAAATTCAAAAATAAAAATGAAAATAATAGATTTAATAAGATAAGAGGTCA  -500

Clark   ATAAAAATTTCTAAAAACGCTTTCATTGAAAAATAAATTCTAAAAAAAA--CACATTCAA  -442
kw4     ATAAAAATTTCTAAAAACGCTTTCATTGAAAAATAAATTCTAAAAAAAAAACACATTCAA  -440

Clark   AAAGAAATTCTAAAAGAAAAGAGAAATAGGTATTATATATTTAATTTACTATCAAAAAAT  -382
kw4     AAAGAAATTCTAAAAGAAAAGAGAAATAGGTATTATATATTTAATTTACTATCAAAAAAT  -381

Clark   CTTTTGAAAATTTAAACTCTTGTTATATTAGTCTTTACAGAAGGCTAATGTAATGCTATA  -322
kw4     CTTTTGAAAATTTAAACTCTTGTTATATTAGTCTTTACAGAAGGCTAATGTAATGCTATA  -321

Clark   ATTGTAATATATTAAATTACTGACAAAAAATTTAAAATATTAGTATCATACACTTAAAGA  -262
kw4     ATTGTAATATATTAAATTACTGACAAAAAAATTAAAATATTAGTATCATACACTTAAAGA  -261

Clark   GACATATTTAAAATCAAATGAATTTAGTTTGATTAGTTAAACATATAATGTATATAATTT  -202
kw4     GACATATTTAAAATCAAATGAATTTAGTTTGATTAGTTAAACATATAATGTATATAATTT  -201

Clark   AAGAGTATTATAAATCTTTTAATAATGTGTTGAATTCTTACCAATAAACAATAAAAAGGA  -142
kw4     AAGAGTATTATAAATCTTTTAATAATGTGTTGAATTCTTACCAATAAACAATAAAAAGGA  -141

Clark   TATTTAAAAGCTCAGTCCTCTTTGTGCAATTGTTTCTATAAAAGCACCCACTTCATACGG   -82
kw4     TATTTAAAAGCTCAGTCCTCTTTGTGCAATTGTTTCTATAAAAGCACCCACTTCATACGG   -81

Clark   TTTCTTCCATTCCATTTTCAAGCTAAGCCTTATAAATAATAAGAGC-AAAAAAAAAAAAC   -22
kw4     TTTCTTCCATTCCATTTTCAAGCTAAGCCTTATAAATAATAAGAGCCAAAAAAAAAAAA-   -21

Clark   CAAAACAACGAGAGAGAGAACATGGGTTCAAGTTCAGCATCCGAAAGTGTTTGCGTTACA    39
kw4     -AAAACAACGAGAGAGAGAACATGGGTTCAAGTTCAGCATCCGAAAGTGTTTGCGTTACA    39
```
